# A Surface Plasmon Resonance-Based Photonic Crystal Fiber Sensor for Simultaneously Measuring the Refractive Index and Temperature

**DOI:** 10.3390/polym14183893

**Published:** 2022-09-17

**Authors:** Jingao Zhang, Jinhui Yuan, Yuwei Qu, Shi Qiu, Chao Mei, Xian Zhou, Binbin Yan, Qiang Wu, Kuiru Wang, Xinzhu Sang, Chongxiu Yu

**Affiliations:** 1 The State Key Laboratory of Information Photonics and Optical Communications, Beijing University of Posts and Telecommunications, Beijing 100876, China; 2 Research Center for Convergence Networks and Ubiquitous Services, University of Science Technology Beijing, Beijing 100083, China; 3 Department of Physics and Electrical Engineering, Northumbria University, Newcastle upon Tyne NE1 8ST, UK

**Keywords:** photonic crystal fiber, surface plasmon resonance, refractive index sensor, temperature sensor

## Abstract

In this paper, a surface plasmon resonance (SPR)-based photonic crystal fiber (PCF) sensor is proposed for simultaneously measuring the refractive index (RI) and temperature. In the design, the central air hole and external surface of the proposed PCF are coated with gold films, and an air hole is filled with the temperature-sensitive material (TSM). By introducing the inner and outer gold films and TSM, the RI and temperature can be measured simultaneously at different wavelength regions. The simulation results show that the average wavelength sensitivities of the proposed SPR-based PCF sensor can reach 4520 nm/RIU and 4.83 nm/°C in the RI range of 1.35~1.40 and a temperature range of 20~60 °C, respectively. Moreover, because of using the different wavelength regions for sensing, the RI and temperature detections of the proposed SPR-based PCF sensor can be achieved independently. It is believed that the proposed SPR-based PCF RI and temperature sensor has important applications in biomedicine and in environmental science.

## 1. Introduction

Surface plasmon resonance (SPR) is an optical phenomenon caused by collective electronic vibration at the metal–dielectric interface [1,2,3]. When the SPR effect occurs, the concentration of the electromagnetic fields in the medium increases greatly, so that it is highly sensitive to the change of the refractive index (RI) of the surrounding media. Owing to the potential for monitoring changes at the metal interfaces, the SPR effect is widely used in the field of sensing [4,5,6,7]. In recent years, with the emergence of photonic crystal fiber (PCF), it has been found that PCF can be well combined with the SPR effect due to its unique optical characteristics and controllable structures [8,9,10,11]. Many works have been concentrated on utilizing the SPR-based PCF sensor to measure the RI [12], temperature [13], magnetic field [14], strain [15], and biological samples [16].

As the design and fabrication technologies of the PCF develop, different kinds of SPR-based PCF sensors have been proposed. In 2020, Hossain et al. improved the RI sensing performance by introducing a hollow core to interact with the metallic surface coated at the external of the PCF. In this work, a sensitivity of 21,000 nm/RIU was achieved in the RI detection range from 1.33 to 1.42 [17]. In the same year, Qiu et al. proposed an all-solid cladding dual-core PCF filled with toluene and ethanol, whose temperature sensitivity reached −15 nm/°C [18]. In 2022, Zhang et al. proposed a novel SPR-PCF RI sensor that could realize an ultra-wide detection range for both the *x*-polarization and *y*-polarization core mode [19]. However, the single parameter measurement sensor cannot meet some complex scenarios in the practical applications. In addition, the simple cascaded single parameter sensor has serious crosstalk problem between different sensing parameters. Therefore, some researchers have proposed multi-parameter sensors to effectively solve the problem. In 2015, Yin et al. demonstrated a novel scheme for the simultaneous measurement of the temperature and RI by cascading a liquid-filled PCF and a long period fiber grating [20]. It is difficult for the proposed scheme to simplify the structure and improve the sensitivity. In 2019, Liu et al. proposed an RI and a temperature sensor based on the SPR effect in D-shaped PCF. It was demonstrated that the maximum RI and temperature sensitivities of the proposed sensor achieved 44,850 nm/RIU and −16.875 nm/°C, respectively [21]. It is impossible for the proposed sensor to simultaneously measure the RI and temperature because the analyte to be measured cannot be changed. In 2020, Guo et al. proposed a D-shaped SPR-based PCF filled with the liquid crystal for detecting the RI and temperature. The simulation results showed that the proposed sensor had RI and temperature sensitivities of 2275 nm/RIU and 9.09 nm/°C, respectively [22]. For the proposed sensor, the influences of the temperature and RI of the analyte to be measured on the core mode cannot be separated, and so it is difficult to realize the simultaneous measurement of the two parameters.

At present, some works have achieved the simultaneous measurement of different parameters by using the orthogonal polarization states of the core mode at the same wavelength region. In 2021, Danlard et al. designed a quasi D-shape temperature and RI SPR-based PCF sensor coated with gold material and filled with temperature-sensitive material (TSM), where the sensitivity reached 5000 nm/RIU in the RI range from 1.35 to 1.46 for the *y*-pol core mode, as well as 3.0 nm/°C in the temperature range from −50 to 50 °C for the *x*-pol core mode [23]. In 2021, Chen et al. reported highly sensitive detection of the RI and temperature based on the D-shaped SPR-based PCF sensor filled with the selective TSM ethanol. The simulation results showed a sensitivity of 3940 nm/RIU in the RI range of 1.35 to 1.40 for the *y*-pol core mode, and a sensitivity of 1.075 nm/°C in the temperature range of 20 to 60 °C for the *x*-pol core mode were achieved [24]. In previous works, although the RI and temperature can be measured simultaneously, the method of using the orthogonal polarization states cannot completely isolate the crosstalk between the two parameters. Therefore, there is an urgent need to design the multi-parameter sensor, which could realize the simultaneous sensing of different parameters, along with negligible crosstalk.

In this paper, a simple SPR-based PCF sensor that can simultaneously measure the RI and temperature is proposed. The RI and temperature sensing performances of the proposed SPR-based PCF sensor is investigated by using the finite element method (FEM). By introducing the inner and outer gold films and TSM, the RI and temperature can be measured simultaneously at different wavelength regions. Moreover, it can avoid crosstalk between the two parameters and realize independent sensing. The simulation result shows that the average wavelength sensitivity of the proposed sensor can reach 4520 nm/RIU in the RI range of 1.35~1.40 and 4.83 nm/°C in the temperature range of 20~60 °C, respectively. It is believed that the proposed SPR-based PCF RI and temperature sensor has important applications in biomedicine and environmental science.

## 2. Design and Operating Principle of the SPR-Based PCF Sensor 

**Figure 1:**
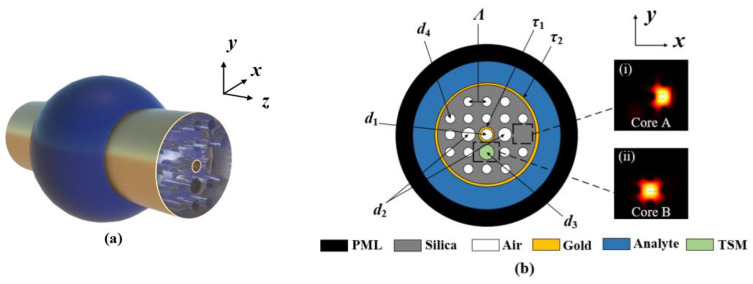
(**a**) The 3D view of the proposed SPR-based PCF sensor. (**b**) Cross-sectional schematic of the SPR-based PCF sensor. The inserts (i) and (ii) show the electric field distributions of the core A and B, respectively.

Figure 1a shows the 3D view of the proposed SPR-based PCF sensor. Figure 1b shows the cross-sectional schematic of the SPR-based PCF sensor, and the inserts (i) and (ii) show the electric field distributions of the cores A and B, which can be used for achieving RI and temperature sensing, respectively. The fiber structure consists of two layers of air holes arranged in a rectangle lattice with a lattice spacing of *Λ*. To excite the SPR effect, the central air hole and the external surface of the proposed PCF are coated with gold films, which are referred to as the inner and outer gold films, respectively. On the *x*-axis side of the outer air holes, an air hole is omitted for forming the core A. Meanwhile, the four corners of the outer air holes are omitted for reducing the distance between the core A and the outer gold film, to enhance the intensity of the evanescent field. The diameter of the central air hole is *d*_1_, and the diameter of the air holes in the first layer along the *x*-axis direction is *d*_2_. On the *y*-axis side of the first layer of air holes, an air hole with the diameter *d*_3_ is filled with the TSM, forming the core B, and the diameter of the other air holes is *d*_4_. The thicknesses of the inner and gold films are *τ*_1_ and *τ*_2_, respectively. A liquid analyte with a thickness of 5 μm is introduced on the outside of the outer gold film. In the simulation, the eigenmode is solved using the FEM, and a perfectly matched layer (PML) is placed to truncate the computational region and to absorb the radiation energy [25,26]. Fused silica is used as the background material, whose material RI can be obtained using the following Sellmeier equation [27,28].

(1) $$n_{{\mathrm{SiO}}_{2}}^{2}=1+\frac{0.6961663\lambda^{2}}{\lambda^{3}-0.0684043}+\frac{0.4079426\lambda^{2}}{\lambda^{2}-0.1162414^{2}}+\frac{0.897479\lambda^{2}}{\lambda^{2}-9.896161^{2}},$$

where *λ* is the free-space wavelength.

The gold is used as a metal material to excite the SPR effect, and the dispersion of the gold film can be obtained via the Lorentz–Drude model, expressed as [29,30]

(2) $$\varepsilon_{m}=\varepsilon_{\infty }-\frac{\omega_{D}^{2}}{\omega \left(\omega -j\gamma_{D}\right)}-\frac{\Delta \varepsilon \cdot \Omega_{L}^{2}}{\left(\omega^{2}-\Omega_{L}^{2}\right)-j\Gamma_{L}\omega },$$

where *ε*_∞_ = 5.9673 represents the high-frequency dielectruc constant, *ω* = 2πc/λ is the angular frequency of the guiding light, *ω*_D_ and *γ*_D_ are the plasma frequency and damping frequency, and Δ*ε* = 1.09 represents the weight factor. Ω_L_ and Γ_L_ represent the frequency and spectral width of the Lorentz oscillator, where Ω_L_/2π = 650.07 THz and Γ_L_/2π = 104.86 THz, respectively.

The TSM is a liquid, whose RI changes with the temperature, the thermal optical coefficient *α* of the TSM is chosen as 3.9 × 10^−4^/°C, and *T* is the temperature in Celsius. The RI of the TSM (*n*_TSM_) can be described as [31].

(3) $$n_{\mathrm{TSM}}=1.454-\alpha \left(T-25\right).$$

It is well known that the SPR-based PCF sensor is dependent on the interaction of the evanescent field with the surface electrons of the plasmonic material [32,33]. The evanescent field originates from the light wave propagated inside the core region of the PCF. When the free electrons on the metal surface are stimulated by the evanescent field, the surface plasma waves (SPWs) are generated. When the phase-matching condition is satisfied, the evanescent wave resonates with the SPWs, and most of the energy of the evanescent wave is transferred to the surface plasmon polariton (SPP) [34,35,36,37,38]. At this time, the propagation loss will appear a sharp peak. When the surrounding medium RI has a slight change, the loss characteristics will be changed significantly. The confinement loss (*CL*) that represents the loss in propagation can be obtained as the following [39,40]

(4) CL(dB/cm)=20ln10×k0×Im(neff)×104,

where *k*_0_ is the free space wave-number and *n*_eff_ is the effective RI of the core mode.

**Figure 2:**
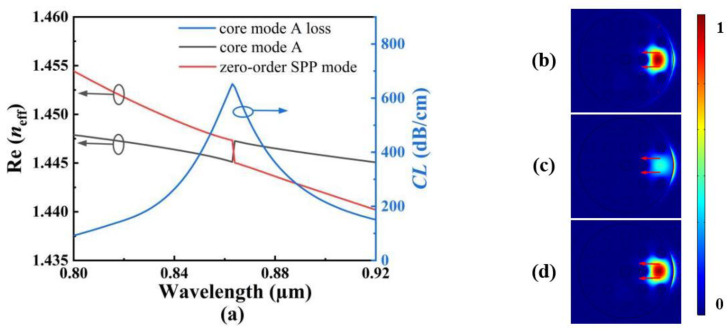
(**a**) The *CL* spectra of the core mode A, and the effective RI real parts of the core mode A and zero-order SPP mode when the analyte RI is chosen as 1.40. (**b**–**d**) The mode field distributions of the core mode A and zero-order SPP mode calculated at wavelengths 0.82, 0.863, and 0.9 μm, respectively.

In this work, by using the inner and outer gold films to excite the SPR effect under different conditions, the proposed SPR-based PCF sensor can simultaneously measure the RI and temperature at different wavelength regions. In order to ensure the conditions of the simultaneous measurement and the flatness of the *CL* spectrum, we select the *x*-pol for sensing. Figure 2a shows the *CL* spectra of the core mode A and the effective RI real parts of the core mode A and zero-order SPP mode when the analyte RI is chosen as 1.40. Figure 2b–d show the mode field distributions of the core mode A and the zero-order SPP mode calculated at wavelengths 0.82, 0.863, and 0.9 μm, respectively. From Figure 2a, the core mode A is coupled with the zero-order SPP mode on the surface of the outer gold film when the wavelength is less than 1 μm, and the *CL* values of the core mode A are small at the non-resonant wavelength. This is because most of energy is confined within the core mode A, as shown in Figure 2b,d. At the resonant wavelength, the phase-matching condition is satisfied, and the corresponding *CL* value of the core mode A reaches the peak. At this time, most of the energy is transferred from the core mode A to the zero-order SPP mode, as shown in Figure 2c.

**Figure 3:**
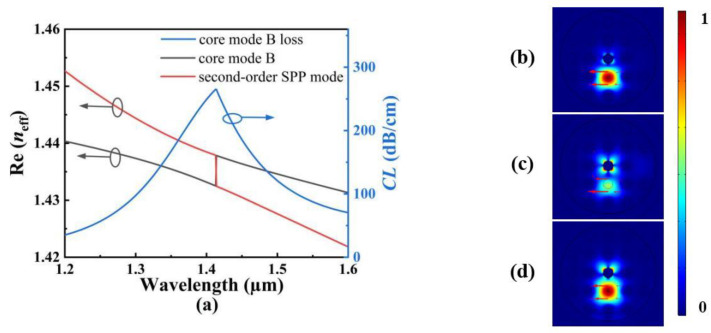
(**a**) The *CL* spectra of the core mode B, and the effective RI real parts of the core mode B and second-order SPP mode when the temperature is chosen as 20 °C. (**b**–**d**) show the mode field distributions of the core mode B and the second-order SPP mode calculated at wavelengths 1.3, 1.414, and 1.5 μm, respectively.

Figure 3a shows the *CL* spectra of the core mode B, and the effective RI real parts of the core mode B and second-order SPP mode when the temperature is chosen as 20 °C. Figure 3b–d show the mode field distributions of the core mode B and second-order SPP mode calculated at wavelengths 1.3, 1.414, and 1.5 μm, respectively. From Figure 3a, the core mode B is coupled with the second-order SPP mode on the surface of the inter-gold film when the wavelength is larger than 1 μm. Similar to the core mode A, at the non-resonant wavelength, the *CL* values of the core mode B are small. This is because most of energy is confined within the core mode B, as shown in Figure 3b,d. At the resonant wavelength, the phase-matching condition is satisfied, and the corresponding *CL* value of the core mode B reaches the peak. At this time, most of energy is transferred from the core mode B to the second-order SPP mode, as shown in Figure 3c. Because the core mode A is coupled with the zero-order SPP mode at the shorter wavelength side and the core mode B is coupled with the second-order SPP mode at the longer wavelength side, the *CL* spectra used for measuring the RI and temperature could be obtained at the same time using a broadband light source. Meanwhile, by comparing the mode field distributions of the core modes A and B at different wavelengths, it is found that there is no cross-coupling when the core modes A and B are coupled with the SPP modes on different gold films. Therefore, the proposed SPR-based PCF sensor can achieve an independent measurement of the RI and temperature.

The change of the analyte RI or temperature will cause the change of the effective RI around the outer or inner gold films, respectively. Thus, the phase-matching condition will change, and the resonant wavelength will occur to shift. At this time the change of the different parameters can be measured by detecting the change of the resonant wavelength. As an important parameter to evaluate the sensing performance, the wavelength sensitivities of the RI and temperature are expressed as [41,42,43].

(5) $$S_{n}\left(\mathrm{nm}/\mathrm{RIU}\right)=\frac{\Delta \lambda_{\mathrm{peak}}}{\Delta n_{a}},$$

(6) $$S_{T}\left(\mathrm{nm}/{}^{\circ}C\right)=\frac{\Delta \lambda_{\mathrm{peak}}}{\Delta T},$$

where Δλ_peak_, Δ*n_a_*, and Δ*T* denote the variations of the resonant wavelength, analyte RI, and temperature, respectively.

## 3. Effects of Structural Parameters on RI and Temperature Sensing

**Figure 4:**
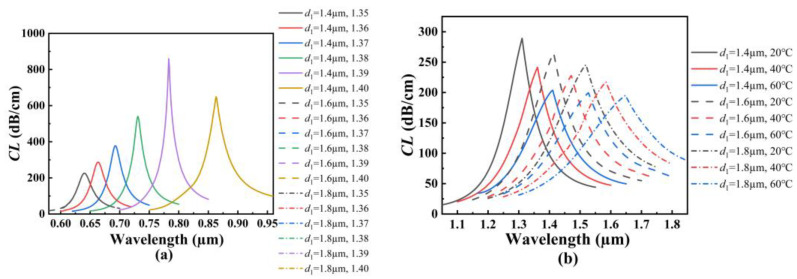
(**a**) The *CL* spectra of the core mode A when *d*_1_ is set as 1.4, 1.6, and 1.8 μm, and when the analyte RI increases from 1.35 to 1.40, respectively. (**b**) The *CL* spectra of the core mode B when *d*_1_ is set as 1.4, 1.6, and 1.8 μm, and when the temperature is chosen as 20, 40, and 60 °C, respectively.

In order to investigate the effects of the structure parameters on the RI and temperature sensing, the initial structure parameters of the proposed SPR-based PCF sensor are set as follows: *d*_1_ = *d*_2_ = *d*_3_ = *d*_4_ = 1.6 μm, *τ*_1_ = *τ*_2_ = 50 nm, and *Λ* = 3 μm. Figure 4a shows the *CL* spectra of the core mode A for different *d*_1_ when the analyte RI increases from 1.35 to 1.40. From Figure 4a, with the increase of the analyte RI, the resonant wavelength of the core mode A occurs to red-shift when *d*_1_ is set as 1.4, 1.6, and 1.8 μm, respectively. It can also be seen from Figure 4a that for the same analyte RI, the resonant wavelength and *CL* peak value do not change, and the average RI sensitivity is also unchanged as *d*_1_ increases. The main reason considered is that the air holes existing around the core A block the interaction between the core A and the fiber core, making the energy well confined within the core mode A. Figure 4b shows the *CL* spectra of the core mode B for different *d*_1_ when the temperature is chosen as 20, 40, and 60 °C, respectively. From Figure 4b, with the increase in the temperature, the resonant wavelength of the core mode B occurs to red-shift when *d*_1_ is set as 1.4, 1.6, and 1.8 μm, respectively. It can also be seen from Figure 4b that for the same temperature, the resonant wavelength occurs to red-shift, and the *CL* peak value slightly decreases with *d*_1_ increases. This may be because the coupling strength between the core mode B and SPP mode becomes gradually weaker as *d*_1_ increases. According to Equation (6), the average temperature sensitivity can reach 2.5, 2.85, and 3.225 nm/°C when *d*_1_ is set as 1.4, 1.6, and 1.8 μm, respectively, which means that the temperature sensitivity of the SPR-based PCF sensor increases with the increase in *d*_1_.

**Figure 5:**
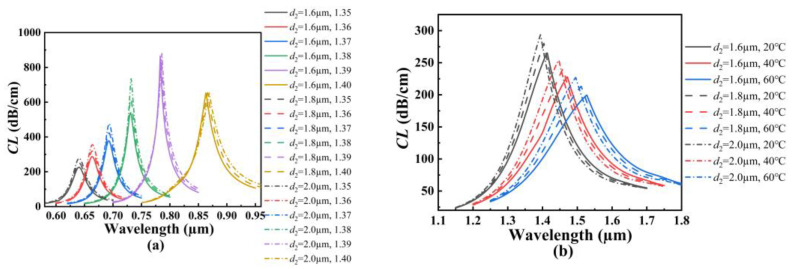
(**a**) The *CL* spectra of the core mode A when *d*_2_ is set as 1.6, 1.8, and 2.0 μm, and when the analyte RI increases from 1.35 to 1.40, respectively. (**b**) The *CL* spectra of the core mode B when *d*_2_ is set as 1.6, 1.8, and 2.0 μm, and the temperature is chosen as 20, 40, and 60 °C, respectively.

Figure 5a shows the *CL* spectra of the core mode A for different *d*_2_ when the analyte RI increases from 1.35 to 1.40. From Figure 5a, with the increase in the analyte RI, the resonant wavelength of the core mode A occurs to red-shift when *d*_2_ is set as 1.6, 1.8, and 2.0 μm, respectively. It can also be seen from Figure 5a that for the same analyte RI, the resonant wavelength occurs to red-shift slightly, and the *CL* peak value increases as *d*_2_ increases. The main reason considered is that the increase in *d*_2_ compresses the core A and enhances the coupling between the core mode A and SPP mode on the outer gold film. According to Equation (5), the average RI sensitivity can reach 4460, 4520, and 4560 nm/RIU when *d*_2_ is set as 1.6, 1.8, and 2.0 μm, respectively, which means that the RI sensitivity of the SPR-based PCF sensor increases with the increase in *d*_2_. Figure 5b shows the *CL* spectra of the core mode B for different *d*_2_ when the temperature is chosen as 20, 40, and 60 °C, respectively. From Figure 5b, with the increase in the temperature, the resonant wavelength of the core mode B occurs to red-shift when *d*_2_ is set as 1.6, 1.8, and 2.0 μm, respectively. It can also be seen from Figure 5b that for the same temperature, the resonant wavelength occurs to blue-shift, and the *CL* peak value increases as *d*_2_ increases. The main reason may be that the increase in *d*_2_ reduces the energy leakage of the fiber core in the *x*-axis direction and enhances the coupling between the inner core mode and SPP mode on the inter-gold film. The calculated average temperature sensitivity can reach 2.85, 2.675, and 2.525 nm/°C when *d*_2_ is set as 1.6, 1.8, and 2.0 μm, respectively, which means that the temperature sensitivity of the SPR-based PCF sensor decreases with the increase in *d*_2_.

**Figure 6:**
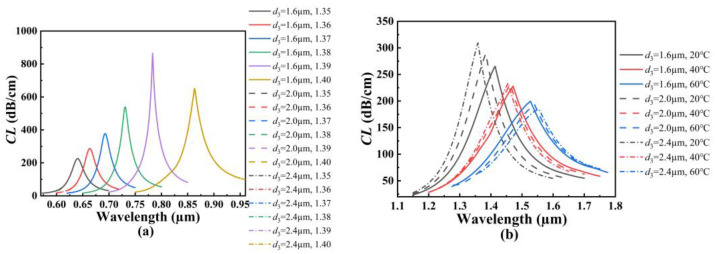
(**a**) The *CL* spectra of the core mode A when *d*_3_ is set as 1.6, 2.0, and 2.4 μm, and the analyte RI increases from 1.35 to 1.40, respectively. (**b**) The *CL* spectra of the core mode B when *d*_3_ is set as 1.6, 2.0, and 2.4 μm, and when the temperature is chosen as 20, 40, and 60 °C, respectively.

Figure 6a shows the *CL* spectra of the core mode A for different *d*_3_ when the analyte RI increases from 1.35 to 1.40. From Figure 6a, with the increase in the analyte RI, the resonant wavelength of the core mode A occurs to red-shift when *d*_3_ is set as 1.6, 2.0, and 2.4 μm, respectively. It can also be seen from Figure 6a that for the same analyte RI, the resonant wavelength and *CL* peak value do not change, and thus, the average RI sensitivity is also unchanged as *d*_3_ increases. The main reason considered is that the air holes that exist around the core A block the effect of the RI change near the fiber core on the outer gold film. Figure 6b shows the *CL* spectra of the core mode B for different *d*_3_ when the temperature is chosen as 20, 40, and 60 °C, respectively. From Figure 6b, with an increase in temperature, the resonant wavelength of the core mode B occurs to red-shift when *d*_3_ is set as 1.6, 2.0, and 2.4 μm, respectively. It can also be seen from Figure 6b that for the same temperature, the resonant wavelength occurs to blue-shift when the temperature is chosen as 20 °C and 40 °C, and the resonant wavelength occurs to red-shift when the temperature is fixed at 60 °C. The main reason is considered as the following: according to Equation (3), the RI of the TSM will decrease as the temperature increases. This means that when the temperature is fixed at 50 °C, the RI of the TSM is equal to that of SiO_2_ at the considered wavelength. Both the RIs of the TSM and SiO_2_ affect the effective RI of the core mode B and the phase-matching conditions. Therefore, the shifts of the *CL* spectra are different when the temperature is greater than 50 °C and less than 50 °C. At this time, the increase in *d*_3_ will enhance the effect of the RI of the TSM on the core mode B. The calculated average temperature sensitivity can reach 2.85, 3.925, and 4.925 nm/°C when *d*_3_ is set as 1.6, 2.0, and 2.4 μm, respectively, which means that the temperature sensitivity of the SPR-based PCF sensor increases with the increase in *d*_3_.

**Figure 7:**
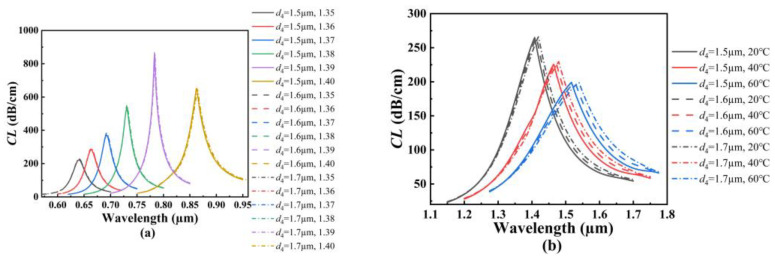
(**a**) The *CL* spectra of the core mode A when *d*_4_ is set as 1.5, 1.6, and 1.7 μm, and when the analyte RI increases from 1.35 to 1.40, respectively. (**b**) The *CL* spectra of the core mode B when *d*_4_ is set as 1.5, 1.6, and 1.7 μm, and when the temperature is chosen as 20, 40, and 60 °C, respectively.

Figure 7a shows the *CL* spectra of the core mode A for different *d*_4_ when the analyte RI increases from 1.35 to 1.40. From Figure 7a, with the increase in the analyte RI, the resonant wavelength of the core mode A occurs to red-shift when *d*_4_ is set as 1.5, 1.6, and 1.7 μm, respectively. It can also be seen from Figure 7a that for the same analyte RI, the resonant wavelength and *CL* peak value changes little, and the average RI sensitivity has almost no change as *d*_4_ increases. The main reason considered is that the increase in *d*_4_ will limit the energy leakage from the core mode A. Thus, the coupling between the core mode A and SPP mode on the outer gold film is not affected. Figure 7b shows the *CL* spectra of the core mode B for different d4 when the temperature is chosen as 20, 40, and 60 °C, respectively. From Figure 7b, with the increase in the temperature, the resonant wavelength of the core mode B occurs to red-shift when *d*_4_ is set as 1.5, 1.6, and 1.7 μm, respectively. It can also be seen from Figure 7b that for the same temperature, the resonant wavelength occurs to red-shift, and the *CL* peak value is unchanged as *d*_4_ increases. This may be because the increase in *d*_4_ changes the effective RI around the fiber core, thus causing the changes of the phase-matching conditions and resonant wavelength. The calculated average temperature sensitivity can reach 2.725, 2.85, and 2.975 nm/°C when *d*_4_ is set as 1.6, 1.8, and 2.0 μm, respectively, which means that the temperature sensitivity of the SPR-based PCF sensor slightly increases with the increase in *d*_4_.

**Figure 8:**
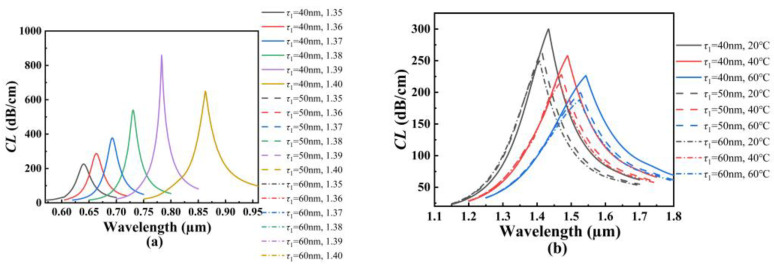
(**a**) The *CL* spectra of the core mode A when *τ*_1_ is set as 40, 50, and 60 nm, and when the analyte RI increases from 1.35 to 1.40, respectively. (**b**) The *CL* spectra of the core mode B when *τ*_1_ is set as 40, 50, and 60 nm, and when the temperature is chosen as 20, 40, and 60 °C, respectively.

Figure 8a shows the *CL* spectra of the core mode A for different *τ*_1_ when the analyte RI increases from 1.35 to 1.40. From Figure 8a, with the increase in the analyte RI, the resonant wavelength of the core mode A occurs to red-shift when *τ*_1_ is set as 40, 50, and 60 nm, respectively. It can also be seen from Figure 8a that for the same analyte RI, the resonant wavelength, *CL* peak value, and average RI sensitivity do not change with the increase in *τ*_1_. The main reason considered is that the air holes existing around the fiber core limit the energy leakage, so that the coupling of the core mode A is not affected by the thickness change of the inner gold film. Figure 8b shows the *CL* spectra of the core mode B for different *τ*_1_ when the temperature is chosen as 20, 40, and 60 °C, respectively. From Figure 8b, with the increase in the temperature, the resonant wavelength of the core mode B occurs to red-shift when *τ*_1_ is set as 40, 50, and 60 nm, respectively. It can also be seen from Figure 8b that for the same temperature, the resonant wavelength occurs to blue-shift, and the *CL* peak value decreases as *τ*_1_ increases. This may be because the increase in *τ*_1_ inhibits the generation of the SPP mode, and the coupling strength between the core mode B and SPP mode on the inner gold film becomes weaker. The calculated average temperature sensitivity can reach 2.725, 2.85, and 2.925 nm/°C when *τ*_1_ is set as 40, 50, and 60 nm, respectively, which means that the temperature sensitivity of the SPR-based PCF sensor slightly increases with the increase in *τ*_1_.

**Figure 9:**
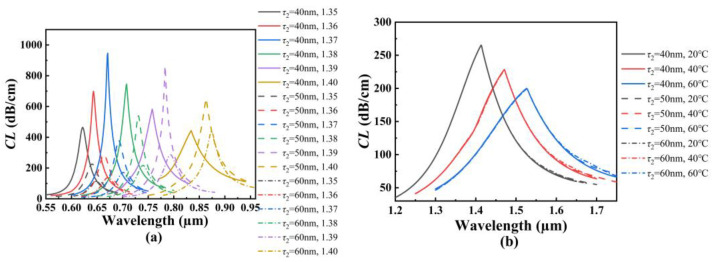
(**a**) The *CL* spectra of the core mode A when *τ*_1_ is set as 40, 50, and 60 nm, and when the analyte RI increases from 1.35 to 1.40, respectively. (**b**) The *CL* spectra of the core mode B when *τ*_1_ is set as 40, 50, and 60 nm, and when the temperature is chosen as 20, 40, and 60 °C, respectively.

Figure 9a shows the *CL* spectra of the core mode A for different *τ*_2_ when the analyte RI increases from 1.35 to 1.40. From Figure 9a, with the increase in the analyte RI, the resonant wavelength of the core mode A occurs to red-shift when *τ*_2_ is set as 40, 50, and 60 nm, respectively. It can also be seen from Figure 9a that for the same analyte RI, the resonant wavelength occurs to red-shift with the increase in *τ*_2_. The *CL* peak value will decrease when the RI is below 1.39, and firstly increase and then decrease when the RI is above 1.39. The main reason considered is that the analyte RI outside and the effective RI inside the fiber affect the coupling between the core mode A and SPP mode on the outer gold film. The calculated average RI sensitivity can reach 4240, 4460, and 4520 nm/RIU when *τ*_2_ is set as 40, 50, and 60 nm, respectively, which means that the RI sensitivity of the SPR-based PCF sensor slightly increases with the increase in *τ*_2_. Figure 9b shows the *CL* spectra of the core mode B for different *τ*_2_ when the temperature is chosen as 20, 40, and 60 °C, respectively. From Figure 9b, with the increase in the temperature, the resonant wavelength of the core mode B occurs to red-shift when *τ*_2_ is set as 40, 50, and 60 nm, respectively. It can also be seen from Figure 9b that for the same temperature, the resonant wavelength, *CL* peak value, and the average temperature sensitivity are unchanged as *τ*_2_ increases. However, the *CL* value slightly increases with the increase in *τ*_2_ when the wavelength is greater than 1.6 μm. This may be because the arrangement of the air holes binds the light inside the fiber core, and because the change of the outer gold film does not affect the coupling between the core mode B and SPP mode on the inner gold film. However, with the increase in wavelength, the limitation of the cladding holes on the energy of fiber core is weakened, resulting in the increase in the *CL* value. The effects of the structural parameters of the proposed SPR-based PCF sensor on the sensing are summarized in Table 1.

## 4. Sensing Performances

By analyzing the variation of the resonance wavelength and mode field, we can draw relevant conclusions, as shown in the table above. However, the above conclusions are obtained when the initial structure parameters are considered as the invariants, and the influences of several structural parameters may make the conclusions localized. Therefore, under comprehensive consideration and calculation, we optimize the structural parameters of the proposed SPR-based PCF sensor as follows: *d*_1_ = 1.7 μm, *d*_2_ = 1.8 μm, *d*_3_ = 2.4 μm, *d*_4_ = 1.6 μm, *τ*_1_ = *τ*_2_ = 50 nm, and *Λ* = 3 μm. In the following, we will discuss the sensing performances of the proposed SPR-based PCF sensor.

### 4.1. Sensitivity, Resolution, and Figure of Merit of the Proposed SPR-Based PCF Sensor

**Figure 10:**
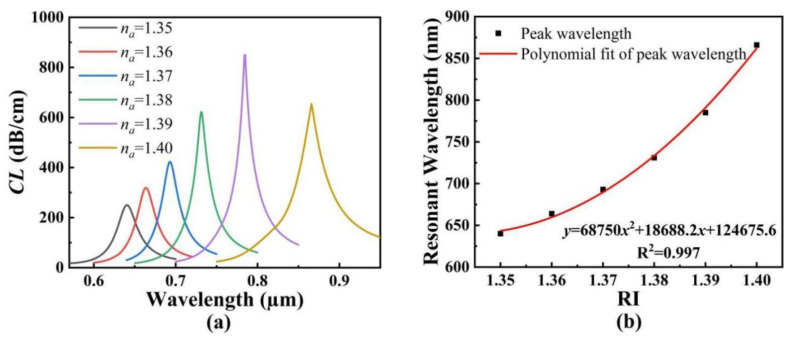
(**a**) The *CL* spectra of the core mode A. (**b**) The variation of the resonant wavelength of the core mode A with the analyte RI and its fitting result.

Figure 10a shows the *CL* spectra of the core mode A when the analyte RI changes from 1.35 to 1.40. From Figure 10a, with the increase in the analyte RI, the *CL* spectra of the core mode A occurs to red-shift, and the corresponding *CL* peak values increase first and then decrease with the increasing wavelength. Figure 10b shows the variation of the resonant wavelength of the core mode A with the analyte RI and its fitting result. As seen from Figure 10b, the relationship equation between the resonant wavelength and analyte RI is *y* = 668,750*x*^2^ + 18,688.2*x* + 124,675.6. The resonant wavelengths change from 640 to 664, to 693, to 731, to 785, and to 866 μm when the analyte RI increases from 1.35 to 1.36, to 1.37, to 1.38, to 1.39, and to 1.40, respectively. The corresponding RI sensitivities are calculated as 2400, 2900, 3800, 5400, and 8100 nm/RIU, respectively, in the RI ranges of 1.35~1.36, 1.36~1.37, 1.37~1.38, 1.38~1.39, and 1.39~1.40. Additionally, the average RI sensitivity is calculated as 4520 nm/RIU.

**Figure 11:**
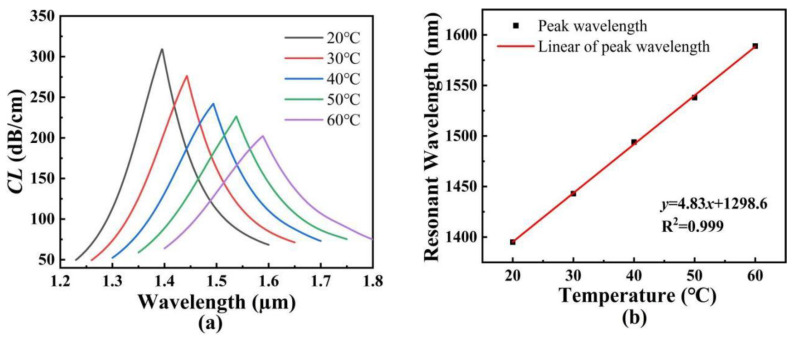
(**a**) The *CL* spectra of the core mode B. (**b**) The variation of the resonant wavelength of the core mode B with the temperature, and its fitting result.

Figure 11a shows the *CL* spectra of the core mode B when the temperature changes from 20 to 60 °C. It can be seen from Figure 11a that with an increase in the temperature, the *CL* spectra of the core mode B occurs to red-shift, and the corresponding *CL* peak values decrease with the increasing wavelength. Figure 11b shows the variation of the resonant wavelength of the core mode B with the temperature and its fitting result. As seen from Figure 11b, the linear fitting result between the resonant wavelength and analyte RI is *y* = 4.83*x* + 1298.6, which has good linearity. The resonant wavelengths vary from 1395 to 1443, to 1494, to 1538, and to 1589 nm when the temperatures increases from 20 to 30, to 40, to 50, and to 60 °C, respectively. The corresponding temperature sensitivities are calculated as 4.8, 5.1, 4.4, and 5.1 nm/°C, respectively, in the temperature ranges of 20~30, 30~40, 40~50, and 50~60 °C. Additionally, the average temperature sensitivity is calculated as 4.83 nm/°C.

In other works, different orthogonal polarization states of the core mode are used to measure the temperature and RI simultaneously. The proposed SPR-based PCF sensor can measure the RI and temperature separately at different wavelength regions, so that it realizes the simultaneous measurement of the two parameters, alone with the negligible crosstalk. Table 2 shows the comparison results of the wavelength sensitivities of the proposed SPR-based PCF sensor with other works.

**Figure 12:**
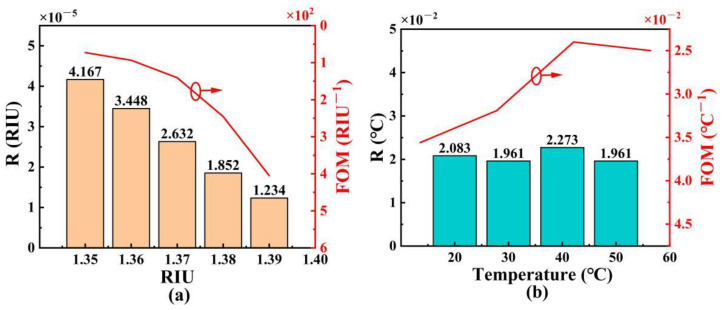
The R and FOM of the proposed SPR-based PCF sensor with the changes of RI (**a**) and temperature (**b**).

Except for the sensitivity, we use the resolution (R) and figure of merit (FOM) to evaluate the sensing performance of the proposed SPR-based PCF sensor [45]. Figure 12a,b show the R and FOM of the proposed SPR-based PCF sensor with the changes of RI and temperature. From Figure 12a, the R shows the decreasing trend as the analyte RI increases, and it is larger than 10^−5^ in the RI range of 1.35~1.40. Additionally, the FOM shows an increasing trend as the analyte RI increases, and the maximum R and FOM of the proposed SPR-based PCF sensor are 1.234 × 10^−5^ RIU and 405 RIU^−1^, respectively. From Figure 12b, as the temperature increases, the R is almost unchanged, and FOM shows the decreasing trend. The maximum R and FOM of the proposed SPR-based PCF sensor are 1.961 × 10^−2^ °C and 3.56 × 10^−2^ °C^−1^, respectively. In summary, the proposed SPR-based PCF sensor can achieve high sensitivity, good linearity, high R, and large FOM for both the RI and temperature sensing at the same time.

### 4.2. Independent Sensing of the Proposed SPR-Based PCF Sensor

**Figure 13:**
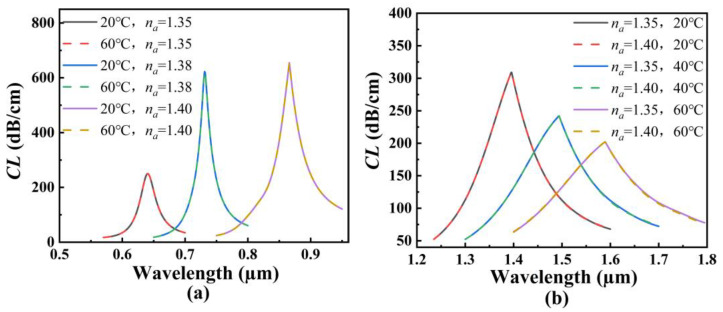
(**a**) The *CL* spectra of the core mode A when the analyte RI is chosen as 1.35, 1.38, and 1.40, and when the temperature is changed from 20 to 60 °C. (**b**) The *CL* spectra of the core mode B when the temperature is chosen as 20, 40, and 60 °C, and when the analyte RI is changed from 1.35 to 1.40.

Figure 13a shows the *CL* spectra of the core mode A when the analyte RI is chosen as 1.35, 1.38, and 1.40, and the temperature is changed from 20 to 60 °C. It can be seen from Figure 13a that for the same analyte RI, the change of the temperature has no effect on the resonant wavelength and the *CL* peak value, and the *CL* spectrum is almost overlapped. This means that the change of the temperature has no effect on RI sensing. Figure 13b shows the *CL* spectra of the core mode B when the temperature is chosen as 20, 40, and 60 °C, and when the analyte RI is changed from 1.35 to 1.40. It can be seen from Figure 13b that for the same temperature, the change of the analyte RI has no effect on the resonant wavelength and the *CL* peak value, and the *CL* spectrum is almost overlapped. It means that the change of the analyte RI has no effect on temperature sensing. The main reason considered is that the two core modes A and B have different phase-matching conditions due to the introduction of the inner and outer gold films, which makes the *CL* spectrum used for RI and temperature sensing well separated at different wavelengths. At the same time, by adjusting the structural parameters, the energy coupling between the two core modes A and B is completely avoided. Therefore, the independent sensing of the RI and temperature can be realized.

## 5. Fabrication Processes of the SPR-Based PCF Sensor

**Figure 14:**
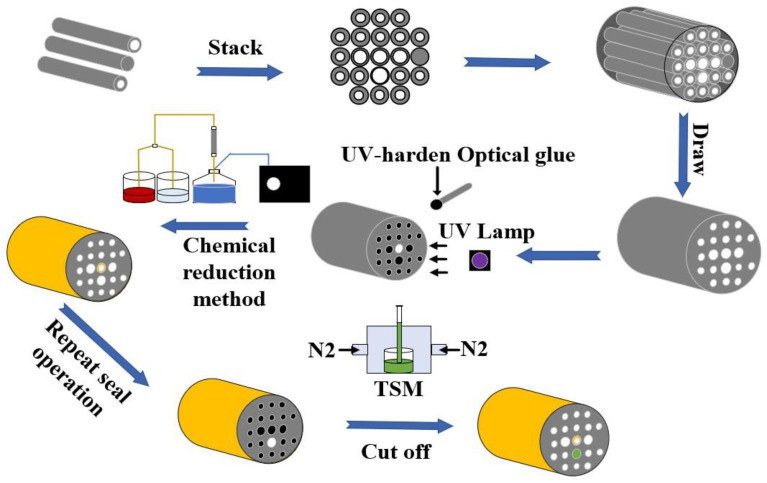
The schematic diagram of the proposed SPR-based PCF sensor fabrication.

Figure 14 shows the schematic diagram of the proposed SPR-based PCF sensor fabrication. The prefabricated rods with different diameters can be easily drawn into the desired structure via the stacking and drawing method [46]. The inner and outer gold films coating inside the central air hole and surface of the PCF can be obtained via the chemical reduction method [47,48]. First, we use the UV glue to block the air holes that do not need to be coated, and we use the UV lamp to irradiate the UV glue. Second, we connect the PCF with the solution containing the metal ions and the reducing agent. Third, through connecting the vacuum pump and adjusting the extraction rate of the vacuum chestnut, the reduced gold can be uniformly attached to the inter-wall of the PCF. Additionally, the outer gold film can be coated in the same way. The TSM can be selectively filled into the air holes using the filling technology [49]. Similarly, the unfilled air holes are sealed with the UV glue, and the TSM is pumped into the air holes of the PCF using a vacuum pump. Finally, the desired fiber length can be obtained by cutting off the fabricated PCF.

This work mainly focuses on the comprehensive structure design and theoretical analysis of the SPR-based PCF sensor, which can be considered as being a valuable guidance for future experimental work. In the next step, we plan to actually fabricate the proposed SPR-based PCF sensor, and experimentally investigate its sensing performances. Moreover, the experimental results will be compared with the theoretical ones to optimize the design and theoretical model.

## 6. Conclusions

In summary, a simple SPR-based PCF sensor that can realize the independent sensing of the RI and temperature is proposed. By introducing the inner and outer gold films and TSM, the RI and temperature can be measured simultaneously at different wavelength regions. The simulation results show that the average wavelength sensitivity of the proposed SPR-based PCF sensor can reach 4520 nm/RIU in the RI range of 1.35~1.40 and 4.83 nm/°C in temperature range of 20~60 °C, respectively, along with negligible crosstalk. The proposed SPR-based PCF sensor is expected to have important applications in biomedicine and in environmental science.

## Figures and Tables

**Table 1:** Effects of the structural parameters of the proposed SPR-based PCF sensor on sensing.

Increase in Structure Parameters	RI		Temperature	
	Resonant Wavelength	Wavelength Sensitivity	Resonant Wavelength	Wavelength Sensitivity
*d*_1_	Unchanged	Unchanged	Red-shift	↑
*d_2_*	Red-shift	↑	Blue-shift	↑
*d*_3_	Unchanged	Unchanged	Red/Blue-shift	↓
*d*_4_	Unchanged	Unchanged	Red-shift	↑
*τ*_1_	Unchanged	Unchanged	Blue-shift	↑
*τ*_2_	Red-shift	↑	Unchanged	Unchanged

Note: ↑: increased, ↓: decreased.

**Table 2:** Comparison results of the sensitivities of the proposed SPR-based PCF sensor with other works.

Operating Wavelength nm	RI Range	Sensitivity nm/RIU	Temperature Range °C	Sensitivity nm/°C	Refs.
1000–1600	1.0–1.6	2275 (Max)	15–50	9.09 (Max)	[21]
1250–1650	1.35–1.46	5000 (Max)	−50–50	3 (Max)	[22]
550–850	1.35–1.40	3940 (Avg)	20–60	1.075 (Avg)	[23]
600–750	1.33–1.34	1371 (Avg)	0–40	1.06 (Avg)	[44]
600–950, 1200–1800	1.35–1.40	4520 (Avg) 8100 (Max)	20–60	4.83 (Avg); 5.1 (Max)	This work

## Data Availability

Not applicable.
